# Bypass of cell cycle arrest induced by transient *DNMT1 *post-transcriptional silencing triggers aneuploidy in human cells

**DOI:** 10.1186/1747-1028-7-2

**Published:** 2012-02-03

**Authors:** Viviana Barra, Tiziana Schillaci, Laura Lentini, Giuseppe Costa, Aldo Di Leonardo

**Affiliations:** 1 Department of Molecular and Biomolecular Science and Technology (STEMBIO) University of Palermo, Italy; 2 Centro di OncoBiologia Sperimentale; Palermo, Italy

**Keywords:** G1 arrest, aneuploidy, DNA methylation, DNMT1

## Abstract

**Background:**

Aneuploidy has been acknowledged as a major source of genomic instability in cancer, and it is often considered the result of chromosome segregation errors including those caused by defects in genes controlling the mitotic spindle assembly, centrosome duplication and cell-cycle checkpoints. Aneuploidy and chromosomal instability has been also correlated with epigenetic alteration, however the molecular basis of this correlation is poorly understood.

**Results:**

To address the functional connection existing between epigenetic changes and aneuploidy, we used RNA-interference to silence the *DNMT1 *gene, encoding for a highly conserved member of the DNA methyl-transferases. DNMT1 depletion slowed down proliferation of near-diploid human tumor cells (HCT116) and triggered G1 arrest in primary human fibroblasts (IMR90), by inducing p53 stabilization and, in turn, p21^waf1 ^transactivation. Remarkably, p53 increase was not caused by DNA damage and was not observed after p14-ARF post-transcriptional silencing. Interestingly, DNMT1 silenced cells with p53 or p14-ARF depleted did not arrest in G1 but, instead, underwent DNA hypomethylation and became aneuploid.

**Conclusion:**

Our results suggest that DNMT1 depletion triggers a p14ARF/p53 dependent cell cycle arrest to counteract the aneuploidy induced by changes in DNA methylation.

## Background

Genomic instability is a characteristic of the majority of human tumors and is considered a driving force for tumorigenesis. Various forms of genome instability have been described and characterized by an increased rate of a number of different genetic alterations [1,2]. Most cancers show a form that is called chromosomal instability (CIN), which refers to the high rate of numerical and structural chromosome changes found in cancer cells compared to normal cells. Numerical CIN is characterized by gains and losses of whole chromosomes (aneuploidy) during cell proliferation. Mutations in genes encoding mitotic regulators [3] and in genes controlling centrosome numbers and tumor suppressors [4-7] have been suggested as molecular defects underlying aneuploidy.

Nevertheless, correct chromosome structure and function may play a role in the stabilization and normal functioning of chromosome segregation. Indeed, it is now accepted that gene expression and chromosome organization are mainly affected by epigenetic marks and may be implicated in the normal chromosome segregation process. Thus, epigenetic alterations should be considered as a cause of aneuploid cells generation [8]. In fact, imbalance in cytosine methylation of CpG islands is a recurrent event in human sporadic cancers. Hypomethylation and hypermethylation occur at specific but different sites within the cancer cell genome and can precede malignancy. Global genome hypomethylation in breast, ovarian, cervical and brain tumors increases with increasing malignancy [9]. However, it is still object of investigation the mechanism(s) that correlates hypomethylation with tumor initiation-progression. Several hypotheses have been proposed, including chromosomal instability induced by hypomethylation of pericentromeric regions [10].

DNA-methyltransferases (DNMTs) - namely DNMT1, DNMT3a and DNMT3b in humans - are directly involved in DNA methylation. DNMT1 differs from the other two human DNA methylases, DNMT3a and DNMT3b, mainly because it is unable to methylate DNA with both strands unmethylated (*de novo *methylation) [11]. DNMT1 is able to restore DNA methylation patterns during S-phase of the cell cycle and it has been recently implicated in genomic stability [12]. In addition, DNMT1 was found deregulated in different human tumors suggesting its involvement in tumor initiation/progression. To investigate DNMT1 implication in the generation of chromosomal instability (aneuploidy), we evaluated the effects of its depletion by RNA interference in primary human fibroblasts (IMR90) and in near diploid human tumor cells (HCT116). *DNMT1 *post-transcriptional silencing in IMR90 cells resulted in G1 arrest, associated with increased expression of p21^WAF1-Cip1 ^and p53 stabilization. p53 stabilization was not caused by DNA damage. Simultaneous p14ARF and DNMT1 transcriptional silencing in IMR90 cells did not result in p53 stabilization and G1 arrest. Accordingly, HCT116 cells, that are p14ARF-null [13] did not arrest in response to DNMT1-depletion. Thus, overriding the G1 arrest after DNMT1-depletion resulted in global DNA hypomethylation and aneuploidy.

Our results suggest that DNMT1 deficiency induces different outcomes depending on the genetic background of the cells. In IMR90 cells DNMT1 depletion resulted primarily in cell cycle arrest, while in HCT116 tumor cells, missing p14ARF, induced aneuploidy, probably affecting the correct chromosomal segregation by altering the DNA methylation pattern.

## Results

### *DNMT1 *depletion induces growth delay in HCT116 cells and G1 arrest in IMR90 cells

**Figure 1:**
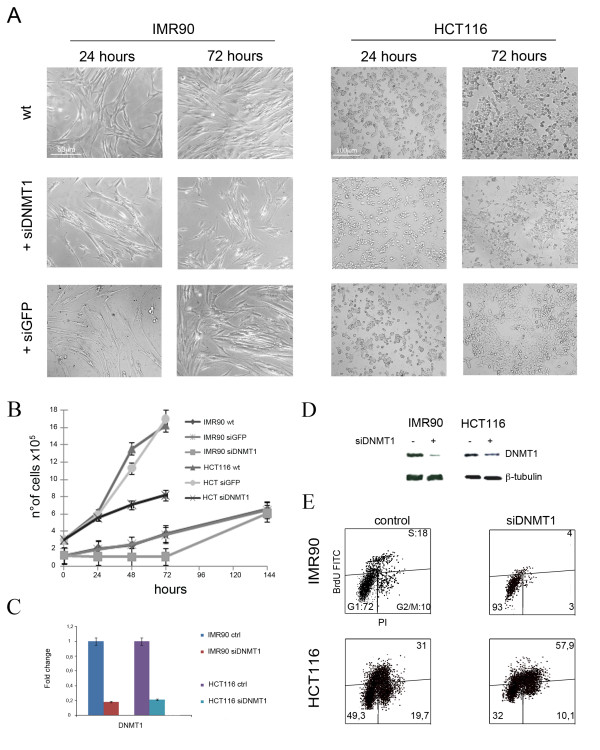
**DNMT1-depletion caused cell growth delay**. A) Morphology of IMR90 and HCT116 cells 24 and 72 hours post-transfection of siDNMT1 or siGFP. There are only slight differences between cells untreated (wt) and transfected with control siRNA (siGFP), on the contrary the treatment with siDNMT1 altered cell density/dish. B) Pictures and histogram showing the effects of siRNAs targeting *DNMT1 *and controls (wt and siGFP) in IMR90 and HCT116 cells on proliferation. Human fibroblasts slowed down proliferation in comparison to control cells at 72 h; however they resumed proliferation at 144 h. HCT116 cells growth was less affected by *DNMT1 *silencing. C) Real Time RT-PCR analysis of IMR90 and HCT116 cells showing decrease of DNMT1 transcriptional reduction after 72 hours from transfection of siRNAs targeting *DNMT1*. D) Western Blot showing the reduction of DNMT1 protein in both IMR90 and HCT116 cells treated with siDNMT1, consistent with Real Time RT-PCR results (see Additional file 1 for the densitometric analysis) E) Cytofluorimetric profiles of IMR90 and HCT116 cells pulsed with BrdU for 1 h, and stained with anti-BrdU antibody FITC-conjugated and propidium iodide. Following *DNMT1 *silencing IMR90 cells arrested in G1, while HCT116 cells slightly accumulated in S phase.

In order to deplete transiently *DNMT1 *in IMR90 and HCT116 cells, we used an siRNA (siDNMT1) targeting a portion of the *DNMT1 *transcript, previously shown to silence DNMT1 specifically [14]. As a control it was used an siRNA targeting the green fluorescent protein (siGFP). Microscopy observation of both cell lines revealed that the control siRNA did not affect cell morphology/viability in comparison with untreated cells. On the contrary transfection with siDNMT1 induced changes in the morphology and proliferation of cells (Figure 1A). By counting cells every 24 hours following siRNAs transfection, we found that IMR90-siDNMT1 cell numbers did not increase during the first 72 hours of silencing, and cells resumed proliferation at 144 hours post-transfection (Figure 1B). Instead, HCT-siDNMT1 cells do proliferate, even if more slowly than control cells during the 72 hours following transfection of siDNMT1 (Figure 1B). On the contrary, we did not observe significative differences in cell proliferation after control siRNA (siGFP) transfection. Real time RT-PCR showed reduction of *DNMT1 *gene expression levels (about 80%) after siDNMT1 transfection in both cellular types (Figure 1C). The experiment was run by using cDNA generated from mRNA extracted at 72 hours post-transfection from both cell types. To determine whether mRNA decreased levels correlated with DNMT1 protein reduction, protein were extracted from cells transfected with siDNMT1 and from control cells. Western blotting for DNMT1 revealed a large decrease of DNMT1 protein in IMR90 cells and only its partial reduction in HCT116 cells (Figure 1D).

It is conceivable that DNMT1 depletion, causing defects in DNA methylation, will be sensed as a stress signal eliciting a cell cycle response (arrest/delay) in the cell. To address this further, we did cytofluorimetric analyses of IMR90-siDNMT1 and HCT-siDNMT1 cells at 72 hours post-transfection to compare them with control cells (siGFP transfected). To assess the number of cells actively replicating DNA (S-phase cells), cells were labeled with BromodeoxiUridine (BrdU) that is incorporated during DNA synthesis, and were then analyzed simultaneously for BrdU incorporation and propidium iodide (PI) staining. Cytofluorimetric analyses showed a large decrease in the number of IMR90-siDNMT1 S-phase cells (BrdU positive) and accumulation of the cells in the G1-phase (Figure 1E) indicative of a G1 arrest. On the contrary, HCT-siDNMT1 cells did not arrest in G1 but they accumulated in S-phase delaying the G2/M phase entry (Figure 1E). Collectively, these findings highlight a different response to *DNMT1 *depletion of IMR90 human fibroblasts versus HCT116 cells.

### IMR90 cells sense DNMT1 decrease and activate a p53-controlled pathway

**Figure 2:**
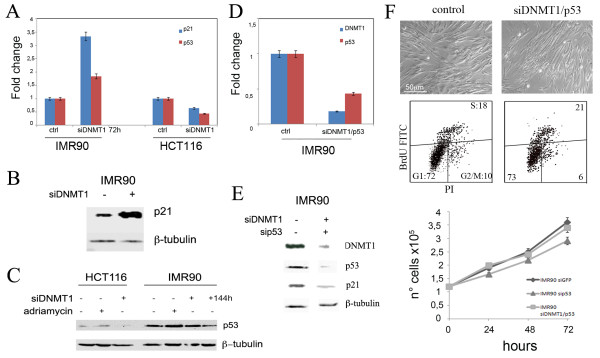
**p53 post-transcriptional silencing abrogates the G1 arrest after DNMT1 depletion**. A) Real time RT-PCR and (B, C) Western Blot analysis revealed a strong increase of p21^waf1-Cip1 ^and p53 protein levels in IMR90 cells and not in HCT116 cells at 72 hours siDNMT1 post-transfection. At 144 hours p53 level was reduced. Cells treated with adriamicyn to induce DNA damage were used to assess the functionality of the p53 pathway. D) Real time RT-PCR and detection of p53 and DNMT1 protein levels by WB (E) in siDNMT1/p53 (72 h) and control IMR90 cells. Summary graphs illustrating differences in p21^waf1-Cip1 ^and p53 protein levels (see Additional files 2, 3, and 4 for the relative densitometric analyses). Data are expressed related to control cells following normalization with β-tubulin. F) Top panels: IMR90-ctrl and IMR90-siDNMT1/p53 cells pictures at 72 hours post-transfection showing that cell density/dish in IMR90-siDNMT1/p53 cells is comparable to that of IMR90-ctrl cells. Central panels: cytofluorimetric profiles showing no significative differences in cell cycle distribution of control and IMR90-siDNMT1/p53 cells. Bottom panel: histogram showing IMR90 siDNMT1/p53 cells proliferation in comparison to control and sip53 cells.

Since DNMT1 is part of the replication fork [15], we reasoned that *DNMT1 *depletion could activate a pathway similar to that triggered by DNA replication stress, leading to inhibition of DNA replication origin firing and thus inducing cell cycle arrest (Figure 1E). To this aim we examined the effect of *DNMT1 *post-transcriptional silencing on p21^WAF1-Cip1 ^expression that is responsible for G1 arrest induced by DNA damage. Real time RT-PCR revealed a strong increase of p21^WAF1-Cip1 ^transcript in IMR90-siDNMT1 cells in comparison to control cells (Figure 2A). These results were confirmed by the increase of both p21^WAF1-Cip1^, and p53 proteins (Figure 2B, C). We observed also increase of p53 transcript levels that was probably caused by the positive feedback of p53 protein on its own promoter, as reported previously [16] (Figure 2A). On the contrary, we did not observe increase of p21^WAF1-Cip1 ^and p53 protein levels in HCT-siDNMT1 cells (Figure 2A, C). Moreover, at 144 hours after *DNMT1 *silencing, when IMR90 cells restart proliferation, we found a reduced amount of the p53 protein (Figure 2C).

These results suggest that p53 might be involved in the G1 arrest observed in IMR90-siDNMT1 cells by transactivating p21^WAF1-Cip1^. To investigate this further, IMR90 cells were simultaneously transfected with siRNAs specifically targeting *DNMT1 *and *p53*. Real time RT-PCR and Western blot analyses confirmed reduced expression of *DNMT1 *and *p53 *in these silenced cells in comparison to control cells (Figure 2D, E). In addition, western blotting showed p21^WAF1-Cip1 ^reduction in these double silenced cells (Figure 2E), indicating its correlation with p53 depletion.

At 72 hours post-transfection the number of double transfected cells was similar to that of control cells (Figure 2F top panel). By counting cells every 24 hours, we noticed that cells proliferate as the control cells (Figure 2F central panel). Cytofluorimetric analyses showed also that siDNMT1/p53 cells progressed normally in the cell cycle, while IMR90-siDNMT1 arrested in G1 (Figures 2F, 1E). These findings suggest that p53 reduction allowed an incorrect cell cycle progression of DNMT1-depleted cells.

**Figure 3:**
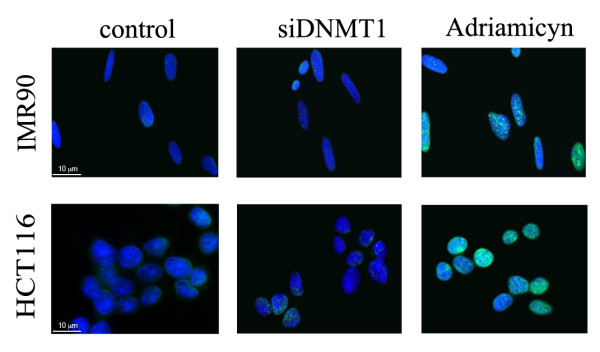
**DNMT1 transient downregulation does not induce DNA damage**. Immunofluorescence analysis against γH2A.X revealed that *DNMT1 *silencing did not induce γH2A.X positive foci formation in both IMR90 and HCT116 cells. Cells treated with Adriamicyn were used as a positive control.

To exclude the presence of DNA damage following DNMT1 depletion we monitored the amount of phosphorylated H2A.X protein by immunofluorescence microscopy. Phosphorylated H2A.X (γH2AX) is a modified histone that accumulates at DNA double-stranded breaks and functions to recruit DNA repair protein. We observed a small difference of γH2AX positive nuclei between control and silenced cells (IMR90-siDNMT1 and HCT-siDNMT1) (Figure 3). On the contrary, Adriamycin treatment increased the percentage of γH2AX positive cells (Figure 3, 60% of observed nuclei) as well the p53 protein level (Figure 2C) indicating that the DNA damage pathway is functional under the our experimental conditions. This result suggests that the cell response pathway activated by DNMT1 depletion, is not induced by DNA damage.

### P14ARF is involved in a cell response to DNMT1 depletion

**Figure 4:**
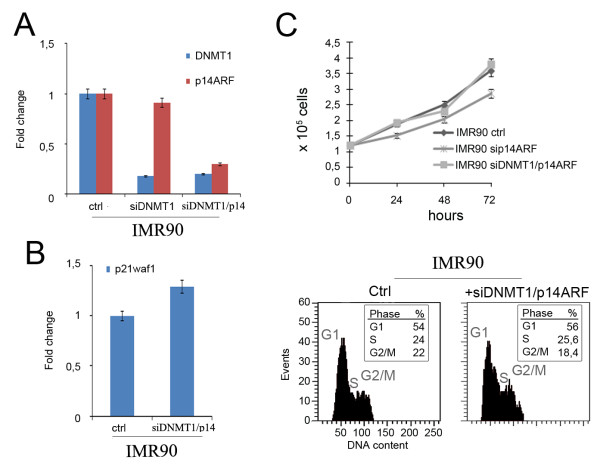
**p14ARF depletion abrogates G1 arrest after DNMT1 depletion**. A) Real time RT-PCR revealed transcript levels of DNMT1 and p14ARF in siDNMT1 and siDNMT1/p14ARF IMR90 cells in comparison with control cells. B) Real time RT-PCR revealed that p21^waf1-Cip1 ^was not transactivated in absence of p14ARF after DNMT1-depletion in IMR90 cells at 72 hours. C) Top panel: histogram showing proliferation of IMR90 siDNMT1/p14ARF cells in comparison to control cells. Bottom panels: cytofluorimetric profiles of IMR90-siDNMT1/p14 cells stained with Propidium Iodide (PI) as indicator of DNA content, which revealed no significative difference on cell cycle progression in comparison to control cells. IMR90-sip14 cells were used also as a control.

The difference between the behaviour of IMR90 and HCT116 cells in response to DNMT1 knock-down seems to be due to a specific cell stress pathway that is missing in HCT116 cells. Since p14ARF, known to stabilize p53 by sequestering MDM2, is not functioning in HCT116 [13], we checked the involvement of p14ARF, in the cellular response to DNMT1 depletion. To this aim we looked at p14ARF transcriptional level by Real time RT-PCR in IMR90-siDNMT1 cells and we did not found significative differences in comparison to control cells (Figure 4A). Nevertheless, to investigate further on p14ARF potential involvement in p53 activation/stabilization after DNMT1 depletion, we performed a double siRNAs transfection with siDNMT1 and sip14ARF in IMR90 cells (Figure 4A). Interestingly, using Real-time RT-PCR we observed that p21^WAF1-Cip1 ^transcripts were not increased (Figure 4B) as observed after single post-transcriptional silencing of DNMT1 (Figure 2A). This result suggested that the p53-dependent pathway triggering the G1 arrest was not activated in these cells. In fact, by counting cells every 24 hours from siRNA transfection, we observed that IMR90 siDNMT1/p14ARF cells proliferated to the same rate of control cells (Figure 4C, top panel). Moreover, 72 hours post-transfection cytofluorimetric analyses showed a cell cycle progression similar to that of control cells (Figure 4C, bottom panels). Altogether, these results suggest that p14ARF is involved in the response of IMR90 cells to DNMT1 knockdown.

### DNMT1 depletion induces a global DNA demethylation

**Figure 5:**
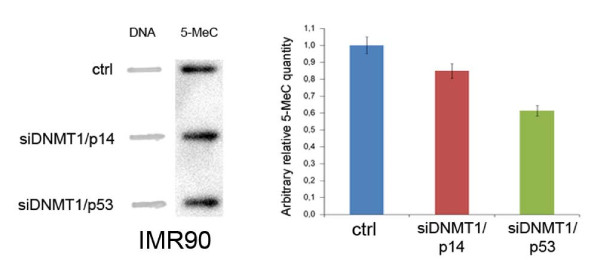
**DNA methylation status in double silenced IMR90 cells**. On the left is shown the Slot-blot analysis of 5-methylcytosine (5-MeC) antibody. DNA samples of control, siDNMT1/p14 and siDNMT1/p53 IMR90 cells were spotted onto Hybond-N membrane and blotted with 5-MeC antibody. Spotted DNA quantity was revealed by methylene blue staining. On the right is shown the graph illustrating differences in 5-MeC quantity between control, siDNMT1/p14 and siDNMT1/p53 IMR90 cells. Data are expressed related to control cells following DNA normalization with methylene blue.

One potential role of the G1-arrest triggered by DNMT1 depletion is to protect the genome from global loss of DNA methylation. DNMT1 has been shown to be loaded on chromatin throughout G2 and M [17], however, it mainly functions as a maintenance DNA-methyltransferase by methylating specific cytosines during DNA replication [18-21]. Thus, the effects of its absence on DNA methylation can be revealed in active proliferating cells. To this aim we investigated if transient *DNMT1 *silencing resulted in DNA demethylation in HCT-siDNMT1 cells that were not G1-arrested. To this aim anti-5-methylcytosine antibody was used to compare the DNA methylation status of HCT-siDNMT1 to HCT116 control cells. Cells were stained with the "capture antibody" (primary) and with the "detection antibody" (secondary) subsequently, and DNA methylation was quantified through an ELISA-like reaction. By this assay the DNA of HCT-siDNMT1 cells resulted globally demethylated (table 1) in comparison to control cells (1,2% DNA methylation vs. 5,65%). By this method, however, we were unable to see differences in DNA methylation between IMR90-siDNMT1/p53, IMR90-siDNMT1/p14, both not G1-arrested (data not shown), and IMR90 control cells. Therefore, we determine DNA methylation by Slot-Blot Analysis using a mouse monoclonal antibody to detect 5-methylcytosine (5-MeC) levels. By this approach we observed reduction of DNA methylation in IMR90-siDNMT1/p53 and IMR90-siDNMT1/p14ARF relative to IMR90 control cells (Figure 5). These results suggest that DNA demethylation can occur in DNMT1-depleted HCT116 and IMR90 cells when they do not arrest in G1

**Table 1:** DNA methylation status after DNMT1 silencing in HCT116

Sample	% Methylated DNA
HCT116 ctrl	5,9% ± 0,4
HCT-siDNMT1	1,95% ± 1,06

DNA extracted from cells was treated with anti-5-methylcytosine antibody, stained with the "capture antibody" and the "detection antibody" and then quantified by an EliSa-like reaction. DNA extracted from HCT-siDNMT1 cells appeared demethylated in comparison to that extracted from HCT116 cells.

### IMR90 and HCT116 cells become aneuploid if they do not arrest in G1 following DNMT1 depletion

**Figure 6:**
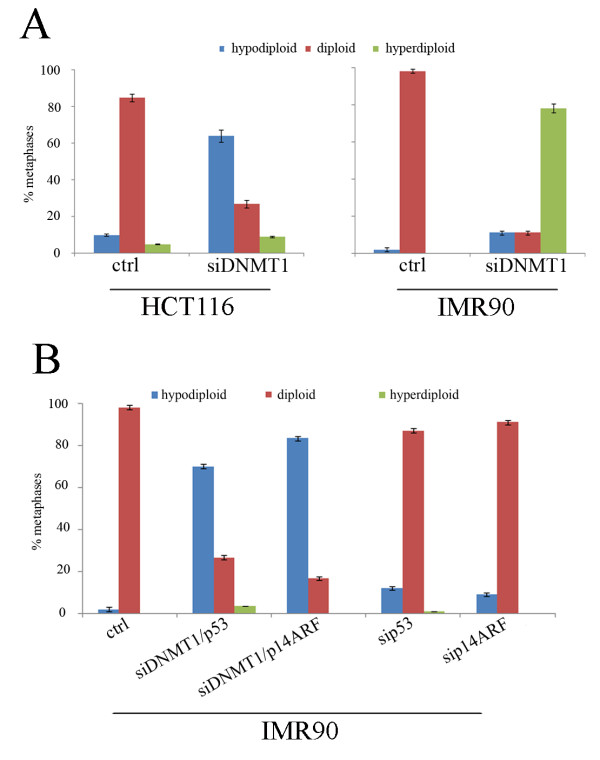
**Cytogenetic effects of transient DNMT1 downregulation**. A) Histograms showing percentages of both aneuploid and diploid metaphases of IMR90-siDNMT1 and HCT-siDNMT1. 35 metaphases were scored for all cell types except that for IMR90-siDNMT1 (n = 11). B) Histograms showing the presence of high number of aneuploid metaphases (mainly hypodiploid) in IMR90-siDNMT1/p53 and -siDNMT1/p14ARF cells at 72 h in comparison to control cells. IMR90 -sip53 and -sip14ARF cells were used as a negative control.

In the last few years it has been hypothesized that DNA hypomethylation in cancer is strictly correlated with the observed genomic instability [22] and in particular with aneuploidy [23-26]. To ascertain if DNA hypomethylation might be implicated in the generation of chromosomal instability, at least in the cells analyzed here, we investigated if transient *DNMT1 *post-transcriptional silencing could induce aneuploidy in diploid cells that did not arrest in G1. To this aim we did cytogenetic analyses in IMR90 and HCT116 cells treated for 72 hours with siDNMT1. The mitotic index of HCT-siDNMT1 and of HCT116 control cells was the same (12%), and the observed mitotic cells (n = 35) were aneuploid (62%, mainly hypodiploid) (Figure 5A, panel on the left). On the contrary, despite of the long colcemid treatment IMR90-siDNMT1 cells showed very few mitotic cells (Mitotic index 0,3% vs 3% of IMR90 control cells) because of G1 arrest, and the majority of them were hyperdiploid (Figure 6A, panel on the right). Thus, the significance of the difference in ploidy between HCT116 and IMR90 cells (hypodiploidy vs hyperdiploidy) has to be considered cautiously.

To ascertain that lack of G1 arrest in presence of decreased DNMT1, as observed in HCT-siDNMT1 cells, contributed to aneuploidy we did a cytogenetic analysis of siDNMT1/p53 and siDNMT1/p14ARF IMR90 cells that despite DNMT1 depletion proliferated incorrectly. This experiment showed that the number of IMR90-siDNMT1/p53 mitotic cells (mitotic index 3%) was similar to that of control IMR90 cells and likely reflects the ability to bypass the G1 arrest. Most importantly the majority (70%) of the observed mitosis (n = 35) were hypodiploid (Figure 6B) as shown by HCT-siDNMT1 cells that did not arrest in G1 (Figure 6A). IMR90-siDNMT1/p14ARF showed the same mitotic index of control cells and were mainly hypodiploid (Figure 6B). These results show that in absence of the G1 arrest provoked by DNMT1 depletion the analyzed cells acquired aneuploidy. This last finding suggests that the G1 arrest is induced to protect cells from aneuploidy likely due to DNA hypomethylation that would occur after DNMT1 depletion.

## Discussion

DNA methylation, histone methylation and deacetylation combine to bring about DNA hypercondensation typical of centromeric and pericentromeric chromatin [27,28]. *DNMT1 *downregulation might alter this combination leading to chromatin decondensation that could determine chromosomes missegregation. Imbalance in cytosine methylation (hypomethylation) and deregulation of DNA methyl-transferases, in particular DNMT1, are recurrent in human sporadic cancers and it seems that they could be involved in the acquisition of chromosomal instability [10]. Deregulated expression of *DNMT1 *is frequently observed in human tumors [12,29-31] however its correlation with cancer progression is still object of investigation.

By using two different cell types, primary human fibroblasts (IMR90) and stable near-diploid tumor cells (HCT116), we determined that DNMT1 transient depletion had different outcomes related to the genetic context of the cell. We observed that the transient *DNMT1 *post-transcriptional silencing promptly induced G1 arrest in human fibroblasts, but only a slight decrease of cell proliferation in HCT116 cells. As a consequence of the proliferation arrest mediated by DNMT1 transient depletion IMR90 fibroblasts showed very few mitotic cells. These findings suggest the existence in IMR90 fibroblasts of a p53 controlled cellular pathway that following DNMT1 depletion activates a putative G1-checkpoint that is not properly working in tumor HCT116 cells. The p53 involvement in response to DNMT1 depletion was further suggested by the finding that double silenced IMR90 cells (IMR90-siDMT1/p53) did not arrest anymore. Our observation that IMR90 cells restarted proliferation after 144 hours from siDNMT1 transfection, and that the p53 amount decreased, are additional evidence of the existence of a p53 controlled checkpoint, that is activated in response to DNMT1 depletion. The absence of γH2AX positive foci rules out that the DNMT1 depletion activates the classic p53 pathway induced by DNA damage. This is confirmed by the fact that HCT116 cells responded correctly to the presence of DNA damage by increasing p53 protein levels, but were unable to halt cellular proliferation in response to transient DNMT1 depletion. Altogether, these results suggest that in HCT116 cells is missing some factor necessary to activate the pathway induced by DNMT1-depletion.

The fact that IMR90-siDNMT1/p14ARF cells did not arrest in G1 indicates that p14ARF is necessary for the cell cycle arrest in IMR90 cells, and also suggests p14ARF as the specific intermediate necessary to activate/stabilize p53 in response to DNMT1 depletion. Thus, the presence of dysfunctional p14ARF in HCT116 cells [13] might explain why these cells cannot properly respond to DNMT1 depletion resulting then in unscheduled progression from G1 to S phase of the cell cycle.

*DNMT1 *silencing and lack of G1 arrest resulted in the induction of aneuploidy in both cell lines analyzed. Recently, it has been proposed that aneuploidy found in different human tumors is caused directly by hypomethylation of specific chromosomal regions that are usually hypermethylated, such as pericentromeric regions, whose function is correlated with chromosome structure (centromere formation) and faithful segregation [23-26]. The incorrect cell cycle progression of HCT116 cells was correlated with a reduction of DNA methylation degree and we hypothesize that DNA hypomethylation could be the cause of aneuploidy in these cells.

Finally, increase in the number of IMR90 aneuploidy cells was seen only after incorrect G1/S progression induced by p53 and p14ARF post-transcriptional silencing. This last finding again highlights the existence of a pathway p14ARF/p53 controlled able to preserve normal cells from incorrect DNA methylation status affected by DNMT1 lack.

## Conclusions

**Figure 7:**
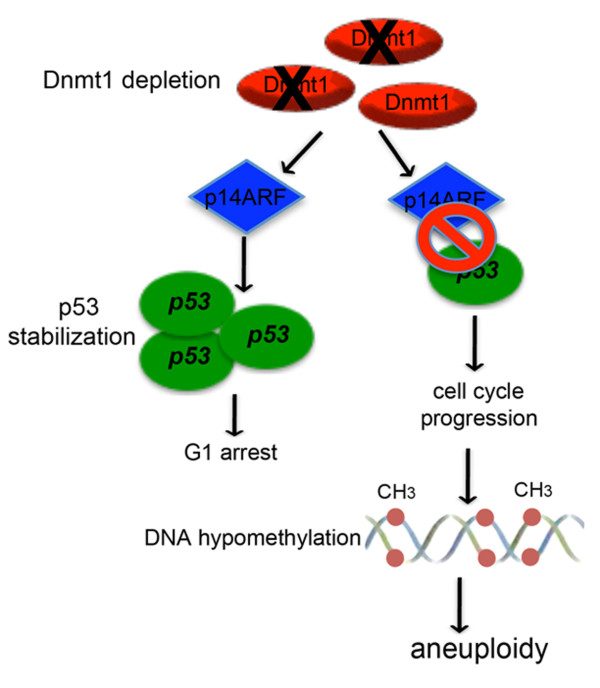
**Speculative model of a putative pathway induced by DNMT1 depletion**. Schematic shows the effects of DNMT1 depletion perceived by a "sensor" factor (p14ARF) that activates a pathway p53-controlled triggering G1 arrest in normal cells. If p53 or p14ARF are absent or not working properly, cells are unable to sense DNMT1 depletion and, thus, progress in the cell cycle incorrectly with wrong DNA methylation patterns triggering aneuploidy.

Altogether our results suggest for the first time the existence of a checkpoint functioning in G1 that protect cells from DNA hypomethylation occurrence that in turn causes aneuploidy. Primary human cells (IMR90) perceive DNMT1 absence that might lead to hypomethylation, as a stress signal and thus, activate a pathway p14ARF/p53 controlled inducing G1 arrest. When this pathway is not working like in HCT116 tumor cells lacking p14ARF or it is abrogated by silencing p14ARF or p53, cells progress incorrectly in the cell cycle with altered DNA methylation pattern (hypomethylation), that might affects also the right chromosomal segregation thus, resulting in aneuploidy (Figure 7). The mechanism by which hypomethylation can influence correct chromosome segregation has to be further studied. Nevertheless, we hypothesize that DNMT1 lack might alter the methylation pattern of pericentromeric chromosomal regions. Hypomethylation may cause chromatin decondensation and in this way to compromise the correct centromere formation, kinethocore assembly, spindle fibres attachment and chromosome segregation.

## Methods

### Cells and cell culture

Human primary fibroblasts (IMR90 passage 12, ATCC) were cultured in EMEM supplemented with: 10% FBS (GIBCO, Life Technologies), 100 units/ml penicillin and 0,1 mg/ml streptomycin (GIBCO, Life Technologies), 1% NEAA (GIBCO, Life Technologies), the human colon cancer cell line HCT116 [32] was cultured in DMEM supplemented with: 10% FBS, 100 units/ml penicillin and 0,1 mg/ml streptomycin. Cells were cultured in a humidified atmosphere of 4% CO_2 _in air at 37°C. For siRNAs transfection 1, 5 × 10^5 ^IMR90 cells and 2, 5 × 10^5 ^HCT116 cells were plated in 6-well dishes and incubated at 37°C. Specific siRNAs duplex were mixed with Lipofectamine2000 Reagent (Invitrogen, Life Technologies), according to manufacturer's recommendation and added to the cells. After 6 hours at 37°C, the transfection medium was replaced with fresh medium. To silence genes of interest post-transcriptionally, cells were transfected with siRNAs targeting *DNMT1 *(siDNMT1: 5'- AUU ACG UAA AGA AGA AUU A dTdT-3') [14] at final concentrations of 60, 80 and 100 nM, siRNAs targeting TP53 (si p53: 5'-GCA UGA ACC GGA GGC CCC AUtt-3') [33] and p14ARF (sip14: 5'-GAA GAU CAG GUC AUG AUG Att-3') [34] at a final concentration of 60 nM. All siRNAs were synthesized by Eurofins-MWG.

### Real time RT-PCR

Primers to be used in Real time RT-PCR experiments were designed with Primer Express software (Applied Biosystems, Life Technologies) choosing amplicons of approximately 70-100 bp. The selected sequences were tested against public databases (BLAST) to confirm the identity of the genes. Total RNA was extracted from cells by using the RNAeasy mini kit (GE) or the "All prep DNA/RNA kit" (Qiagen) according to the manufacture's instruction. RNA was reverse-transcribed in a final volume of 100 μL using the High Capacity c-DNA Archive kit (Applied Biosystems, Life Technologies) for 10 minutes at 25°C and 2 hours at 37°C. For each sample 2 μL of cDNA, corresponding to approximately 100 nag of reverse transcribed RNA, was analyzed by Real time RT-PCR (95°C for 15 sec, 60°C for 60 sec repeated for 40 cycles), in quadruplicate, using the ABI PRISM 7300 instrument (Applied Biosystems). Real time RT-PCR was done in a final volume of 20 μl comprising 1x Master Mix SYBR Green (Applied Biosystems, Life Technologies) and 0, 3 μM of forward and reverse primers for: DNMT1 (Fw: 5'-GCACCTCATTTGCCGAATACA-3'; Rev: 5'-TCTCCTGCATCAGCCCAAATA-3'), TP53 (Fw: 5'-TTCGACATAGTGTGGTGGTGC-3'; Rev: 5'- AGTCAGAGCCAACCTCAGGC-3'), p21^WAF1-Cip1 ^(Fw: 5'-CTGGAGACTCTCAGGGTCGA-3', Rev: 5'-CGGATTAGGGCTTCCTCTTG-3'), p14ARF (Fw: 5'- TGATGCTACTGAGGAGCCAGC-3', Rev: 5'-AGGGCCTTTCCTACCTGGCTC-3'), GAPDH (Fw: 5'-CTCATGACCACAGTCCATGCC-3', Rev: 5'-GCCATCCACAGTCTTCTGGGT-3'). Data were analyzed by averaging quadruplicates Ct (cycle threshold). Levels of RNA expression were determined by using the SDS software version (Applied Biosystems, Life Technologies) according to the 2-ΔΔct method. Levels of RNA expression of selected genes were normalized to the internal control GAPDH.

### Western Blotting

Protein concentration was measured using the Bio-Rad Protein Assay (Bio-Rad Laboratories). Proteins (40 μg) were separated by 10% SDS-PAGE containing 0,1% SDS and transferred to Hybond-C nitrocellulose membranes (Amersham Life Science) by electro-blotting. The membranes were sequentially incubated with goat anti-DNMT1, rabbit anti-p21^WAF1-Cip1^, rabbit anti-pRb (Santa Cruz, CA), mouse anti-p53 (Abcam, UK) as primary antibodies, and HRP-conjugated mouse, rabbit (Abcam, UK) and goat (Santa Cruz, CA) Gig as secondary antibodies. The target protein was detected with enhanced chemiluminescence Western blotting detection reagents (Pierce, Thermo Scientific). We used also mouse anti-β-tubulin (SIGMA, Italy) as internal control of loading. Membranes were stained by Ponca-Red to confirm equivalent loading of total protein in all lanes.

### Cell cycle analysis

Asynchronously growing cells were treated with 80 nM siDNMT1 alone or together with 60 nM sip53 for 72 hours and released into complete medium with BromodeoxiUridine (BrdU, SIGMA, Italy) 0, 2 μg/ml for one hour. DNA content was determined using Propidium Iodide (PI, SIGMA, Italy) staining by treating cells with PBS solution containing 4 μg/ml of PI and 40 μg/ml RNase. Analysis of BrdU labelled cells was conducted as described previously [5] and samples were analyzed on a FACSCanto (Becton Dickinson). Analysis of IMR90-siDNMT1/p14ARF cell cycle was made by PI staining of DNA content. Experiments were repeated at least twice, 10000 events were analyzed by FACSDiva software.

### Cytogenetics analysis

Cells were seeded onto glass cover slides 24 hours before the analysis. They were then, treated with 0,2 μg/ml colcemid (Demecolcine, SIGMA, Italy) for three (HCT116) four (IMR90) or eight (IMR90-siDNMT1) hours. Cell were swollen in 75 mM KCl at 37°C, fixed with methanol/acetic acid (3:1 v/v) The cover slides were then air-dried and stained with 3% GIEMSA in phosphate-buffered saline for 10 minutes. Chromosome metaphases were evaluated using a Zeiss Axioskop microscope under a 63× objective. The analysis was repeated three times for each cell types.

### Immunofluorescence microscopy

*Phosphorylated H2A.X immunostaining*: IMR90 and HCT116 were grown on glass coverslips after siRNAs transfection. Cells on coverslips were fixed with cold methanol (-20°C), permeabilized with 0.01% Triton × (Sigma, Italy) and blocked with 0.1% BSA, both at room temperature. Then, coverslips were incubated with phosphorylated H2A.X rabbit polyclonal antibody (1:100 in PBS-BSA 0.1%, Upstate) overnight at 4°C, washed in PBS and incubated with a FITC-conjugated rabbit anti-mouse (Sigma, Italy) diluted 1:100 in PBS-BSA 0,1%) for 1 hour at 37°C. Nuclei were visualized with 1 μg/ml of 4',6-Diamidino-2-phenylindole (DAPI) and examined on a Zeiss Axioskop microscope (Zeiss, Germany) equipped for fluorescence under a 63× objective. Images were captured with a CCD digital camera (AxioCam, Zeiss).

### Global DNA methylation analysis

"Methylamp Global DNA methylation quantification kit" (Epigentek, USA) was used to quantify global DNA methylation. 200 ng of genomic DNA extracted from cell samples was immobilized in the strip well specifically treated to have high affinity to DNA for 2 hours at 37°C. The methylated fraction of DNA can be recognized by sequential incubation with 5-methylcytosine antibody for 45 minutes at 37°C, and with secondary antibody for 60 minutes at room temperature. A colorimetric reaction allowed methylated DNA quantification at 450 nM with microplate reader. A methylated DNA was used as positive control. The analysis was repeated twice.

### Slot Blot analysis

The DNA-slot blot analysis of 5-Methylcytosine was performed as described [35] with the following modifications. Purified DNA (100 μg/25 μL) from control, siDNMT1/p14ARF and siDNMT1/p53 IMR90 cells, was denatured at 100°C for 5 min and applied onto Hybond-N membrane (RPN203N, Amersham) using the Hybri-slot Manifold Apparatus (Whatman Biometra, Germany) under vacuum for 3 min. DNA was fixed to the membrane by exposing to UV light (90,000 μjoules/cm2) for 45 seconds using the Hoefer UVC 50 Crossliker (Amersham Biosciences). Samples were then treated with TBST, (0.05% Tween20), 5% w/v no-fat dry milk blocking buffer for 1 hour at RT and incubated with 5-Methylcytosine Monoclonal Antibody (1:500, cloneD33, Epigentek USA) in TBST (0.05% Tween20) 5% w/v no-fat dry milk blocking buffer for 1 hour at RT. The membrane was then washed with TBST (0.05% Tween20), 3 times for 10 minutes each, incubated with HRP conjugated secondary antibody anti-mouse (1:2000) (Abcam, UK) in blocking buffer for 1 hour at RT, washed again with TBST (0.05% Tween20), 3 times for 10 minutes each and once in double distilled water for 5 minutes, then developed by enhanced chemiluminescence detection reagents (Pierce Thermo Scientific) and image acquired with Chemidoc XSR Imaging System (BioRad). Spotted DNA quantity was detected by 0.02% w/v methylene blue in 0.3 M sodium acetate (pH5.2) followed by washing in double distilled water to reduce background noise. The relative 5 MeC Optical density (OD) value was calculated with Quantity One 4.6.7 software relative to DNA amount loaded and normalized to wild type sample.

## Competing interests

The authors declare that they have no competing interests.

## Authors' contributions

VB carried out siRNA and cytogenetic experiments, Real time RT-PCR, immunofluorescence microscopy, Western blotting, flow cytometry, participated in the design of the study and drafted the manuscript. TS carried out cytogenetic and proliferation assays. LL carried out Real time RT-PCR, 5MeC-DNA slot-blot experiments and helped to draft the manuscript. GC carried out cell proliferation assays, 5MeC-DNA slot-blot experiments. ADL conceived of the study, participated in its design and coordination and wrote the manuscript. All authors read and approved the final manuscript.

## Supplementary Material

Additional file 1 **Densitometric analysis of Western blot in Figure **1D. Graph illustrating differences in Dnmt1 protein amount between control and siDNMT1 IMR90 cells, and between control and siDNMT1 HCT116 cells. Data are related to control cells following normalization with β-tubulin. Click here for file

Additional file 2 **Densitometric analysis of Western blot in Figure **2B. Graph illustrating differences in p21^waf1 ^protein between control and siDNMT1 IMR90cells. Data are related to control cells following normalization with β-tubulin. Click here for file

Additional file 3 **Densitometric analysis of Western blot in Figure **2C. Graph illustrating differences in p53 protein level between control and siDNMT1 IMR90 cells at 72 h-144 h as well between control and siDNMT1 HCT116 cells at 72 h all treated with adriamicyn. Data are related to control cells following normalization with β-tubulin. Click here for file

Additional file 4 **Densitometric analysis of Western blot in Figure **2E. Graph illustrating differences in DNMT1, p53 and p21^waf1 ^protein levels between control and siDNMT1/p53 MR90 cells. Data are related to control cells following normalization with β-tubulin. Click here for file
