# Supplementary figures and images for: Bypass of cell cycle arrest induced by transient DNMT1 post-transcriptional silencing triggers aneuploidy in human cells
Source: Cell Div. 2012 Feb 3;7:2. doi: 10.1186/1747-1028-7-2 (PMC3292948; doi:10.1186/1747-1028-7-2)

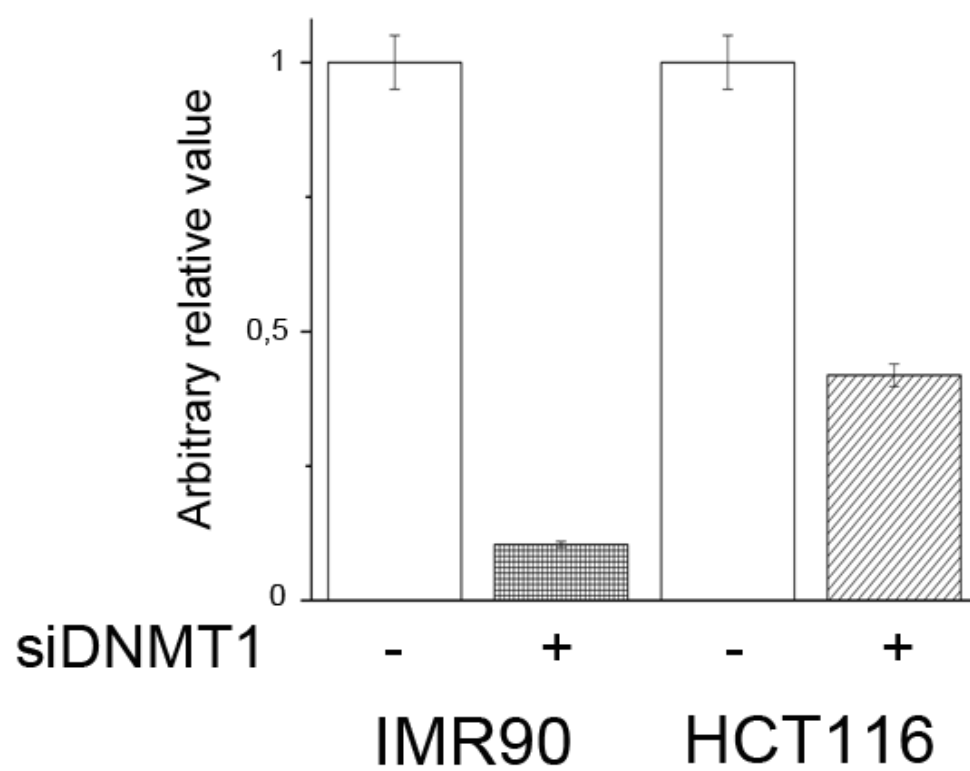

Supplement: Additional file 1 — Densitometric analysis of Western blot in Figure 1D. Graph illustrating differences in Dnmt1 protein amount between control and siDNMT1 IMR90 cells, and between control and siDNMT1 HCT116 cells. Data are related to control cells following normalization with β-tubulin. [file 1747-1028-7-2-S1.PDF]

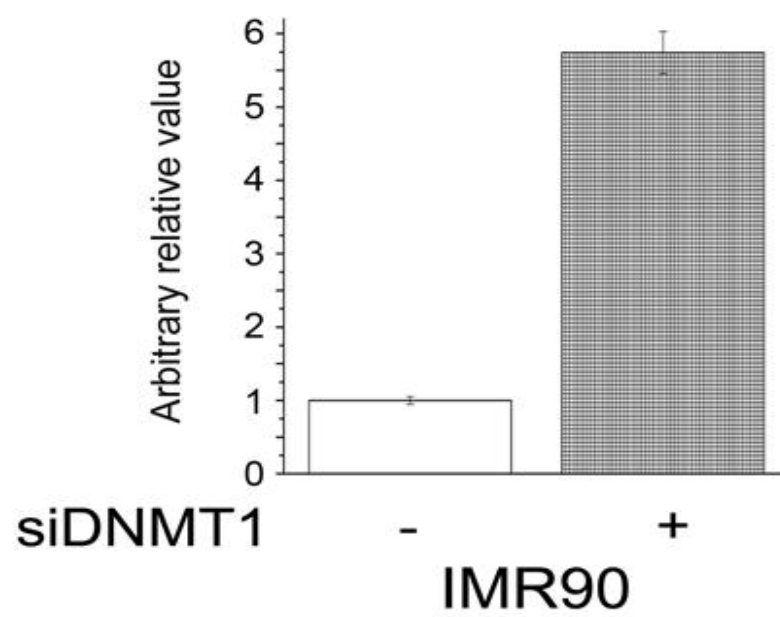

Supplement: Additional file 2 — Densitometric analysis of Western blot in Figure 2B. Graph illustrating differences in p21waf1 protein between control and siDNMT1 IMR90cells. Data are related to control cells following normalization with β-tubulin. [file 1747-1028-7-2-S2.PDF]

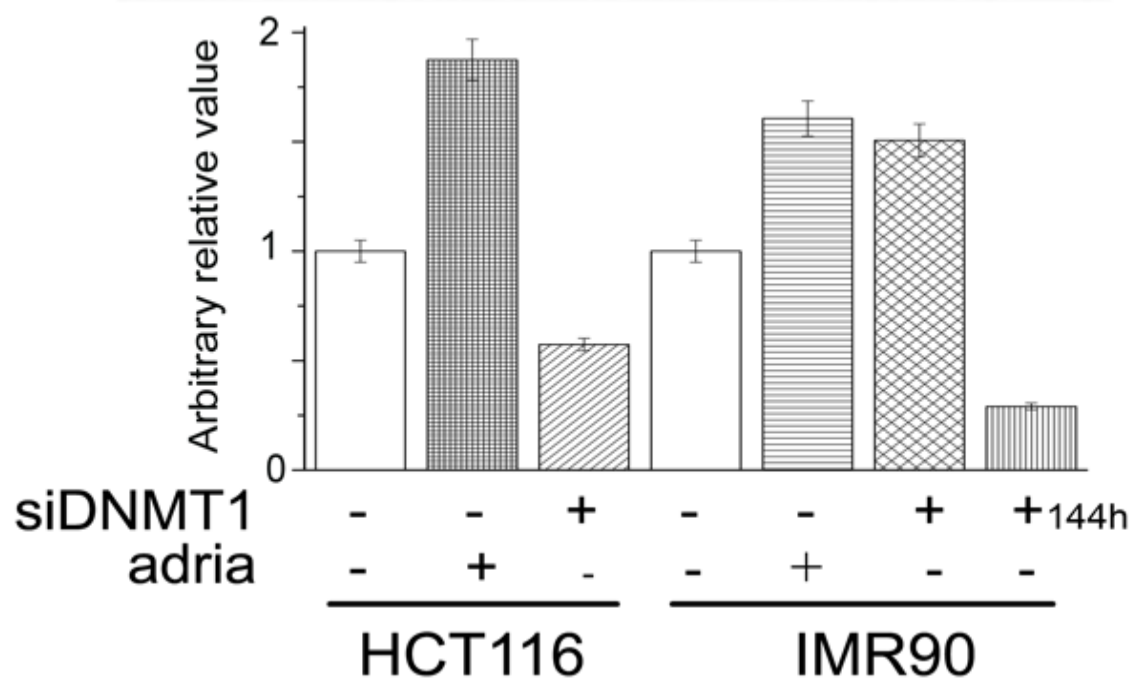

Supplement: Additional file 3 — Densitometric analysis of Western blot in Figure 2C. Graph illustrating differences in p53 protein level between control and siDNMT1 IMR90 cells at 72 h-144 h as well between control and siDNMT1 HCT116 cells at 72 h all treated with adriamicyn. Data are related to control cells following normalization with β-tubulin. [file 1747-1028-7-2-S3.PDF]

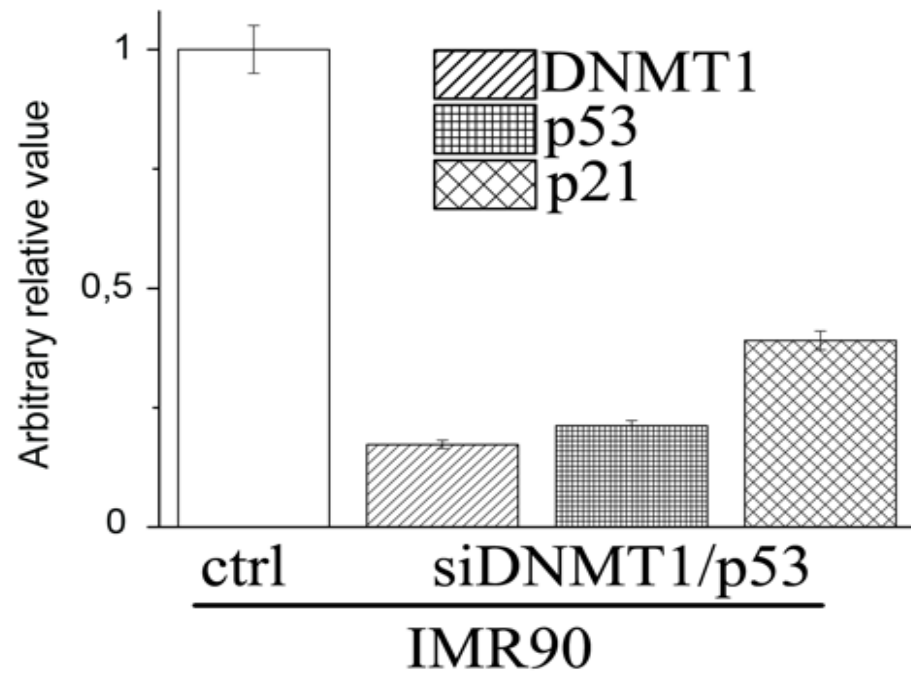

Supplement: Additional file 4 — Densitometric analysis of Western blot in Figure 2E. Graph illustrating differences in DNMT1, p53 and p21waf1 protein levels between control and siDNMT1/p53 MR90 cells. Data are related to control cells following normalization with β-tubulin. [file 1747-1028-7-2-S4.PDF]
